# Dual-energy CT for automatic organs-at-risk segmentation in brain-tumor patients using a multi-atlas and deep-learning approach

**DOI:** 10.1038/s41598-019-40584-9

**Published:** 2019-03-11

**Authors:** Brent van der Heyden, Patrick Wohlfahrt, Daniëlle B. P. Eekers, Christian Richter, Karin Terhaag, Esther G. C. Troost, Frank Verhaegen

**Affiliations:** 1 Department of Radiation Oncology (MAASTRO), GROW—School for Oncology and Developmental Biology, Maastricht University Medical Centre, Maastricht, Netherlands; 2 OncoRay - National Center for Radiation Research in Oncology, Faculty of Medicine and University Hospital Carl Gustav Carus, Technische Universität Dresden, Helmholtz-Zentrum Dresden - Rossendorf, Dresden, Germany; 3 Helmholtz-Zentrum Dresden - Rossendorf, Institute of Radiooncology - OncoRay, Dresden, Germany; 4 Proton Therapy Department South-East Netherlands (ZON-PTC), Maastricht, The Netherlands; 5 Department of Radiotherapy and Radiation Oncology, Faculty of Medicine and University Hospital Carl Gustav Carus, Technische Universität Dresden, Dresden, Germany; 6 German Cancer Consortium (DKTK), partner site Dresden, and German Cancer Research Center (DKFZ), Heidelberg, Germany; 7 National Center for Tumor Diseases (NCT), Partner Site Dresden, Germany: German Cancer Research Center (DKFZ), Heidelberg, Germany; 8 Faculty of Medicine and University Hospital Carl Gustav Carus, Technische Universität Dresden, Dresden, Germany; 9 Helmholtz Association/Helmholtz-Zentrum Dresden - Rossendorf (HZDR), Dresden, Germany

## Abstract

In radiotherapy, computed tomography (CT) datasets are mostly used for radiation treatment planning to achieve a high-conformal tumor coverage while optimally sparing healthy tissue surrounding the tumor, referred to as organs-at-risk (OARs). Based on CT scan and/or magnetic resonance images, OARs have to be manually delineated by clinicians, which is one of the most time-consuming tasks in the clinical workflow. Recent multi-atlas (MA) or deep-learning (DL) based methods aim to improve the clinical routine by an automatic segmentation of OARs on a CT dataset. However, so far no studies investigated the performance of these MA or DL methods on dual-energy CT (DECT) datasets, which have been shown to improve the image quality compared to conventional 120 kVp single-energy CT. In this study, the performance of an in-house developed MA and a DL method (two-step three-dimensional U-net) was quantitatively and qualitatively evaluated on various DECT-derived pseudo-monoenergetic CT datasets ranging from 40 keV to 170 keV. At lower energies, the MA method resulted in more accurate OAR segmentations. Both the qualitative and quantitative metric analysis showed that the DL approach often performed better than the MA method.

## Introduction

In the clinical radiotherapy workflow, the targeted tumor volume and surrounding organs-at-risk (OARs) are manually delineated on image datasets derived from computed tomography (CT), often in combination with magnetic resonance imaging (MRI). In current clinical practice, CT acquisitions are mandatory to calculate radiation treatment plans and to interpret dose evaluation metrics^1,2^. As manual delineation is one of the most time-consuming tasks and subject to inter- and intra-observer variability, a considerable interest in automatic delineation has been seen in recent years to further improve this well-recognized source of uncertainty in the radiation planning process^3–6^. In the last years, multi-atlas (MA) or deep-learning (DL) methods have been investigated for automatic image segmentation^3^. Both approaches use a set of labeled medical image datasets as input for model training and finally application. In this study, CT image datasets with manually delineated OARs serve as reference. Although the fact that automatic contouring algorithms are commercially available, their use in radiotherapy clinics remains limited.

The performance of MA and DL methods for automatic contouring has been already investigated on CT or MRI datasets^3,7,8^. However, to our knowledge, so far no studies explored the use of such methods on dual-energy CT (DECT) image datasets, which provide additional tissue information contributing to a reduction of the intra-observer variability of physicians^9^. DECT scans consist of two CT datasets acquired with different x-ray spectra or energy separation on the detector level. The combined single DECT datasets can be used to calculate a pseudo-monoenergetic image (PMI) with a weighted sum of the low- and high-energy CT scan^10,11^. To enhance the image quality, some commercial systems additionally include noise-suppression algorithms^12^. Furthermore, it has been demonstrated that a PMI can have superior image quality compared to 120 kVp single-energy CT (SECT)^12–15^, which is the current clinical standard in most radiotherapy facilities. The influence of beam hardening on CT numbers can be reduced by PMI datasets leading to improvements in radiation treatment planning, especially in proton therapy^16^. A PMI can also contribute to suppress metal artifacts in CT imaging^10,17^.

This study first aims to quantitatively evaluate pseudo-monoenergetic CT datasets of different photon energies ranging from 40 keV to 170 keV for OAR segmentation in primary brain-tumor patients using an in-house developed 3D MA and 3D DL based image segmentation method. Secondly, two experienced radiation oncologists and one experienced radiation technologist performed a qualitative scoring to assess the clinical relevance and accuracy of automatic OAR segmentation. For this evaluation, both methods were applied on two pseudo-monoenergetic CT datasets of different energy (40 keV and 70 keV).

## Materials and Methods

### Patient cohort and DECT imaging

**Figure 1:**
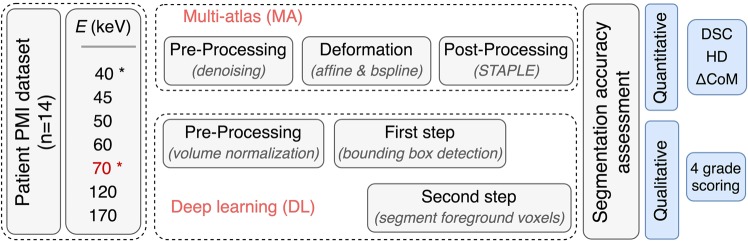
Study flowchart. The multi-atlas (MA) method was applied on all pseudo-monoenergetic image (PMI) datasets. The deep-learning (DL) approach was applied on the energies indicated with an asterisk. The reference energy E (70 keV) was used for manual contouring of the organs-at-risk (OARs). The quantitative and qualitative segmentation accuracy was assessed between the automatically generated contours and the manual contour using the Dice similarity coefficient (DSC), the 95^th^ percentile Hausdorff distance (HD), the center of mass displacement $\triangle {CoM}$ and a four-grade scoring system.

For this retrospective study following the regulatory guidelines and approved by the local ethics committee (EK535122015, University Hospital Carl Gustav Carus, Dresden), 14 primary brain-tumor patients were randomly selected. Each patient agreed with an informed consent to use their pseudonymized and anonymized data for scientific purposes (Fig. 1). All patients underwent DECT imaging (80/140 kVp) for proton treatment planning at University Proton Therapy Dresden (Dresden, Germany) with a single-source DECT scanner (SOMATOM Definition AS, Siemens Healthineers, Forchheim, Germany)^18^. Each DECT scan was acquired with a constant CT dose index (32 cm) of 20.8 mGy and reconstructed using an iterative reconstruction algorithm including a beam hardening correction for bone (SAFIRE, Q34/3 kernel, both Siemens terminology) with a voxel size of 0.98 × 0.98 × 2.00 mm³.

PMI datasets of 7 different energies (40, 45, 50, 60, 70, 120 and 170 keV) were generated from the 80 kVp and 140 kVp DECT scans using the application syngo.CT DE Monoenergetic Plus of the image post-processing software syngo.via (Siemens Healthineers, Forchheim, Germany). The input PMI dataset of the segmentation methods will be referred to as, e.g. PMI-40, i.e. the PMI dataset calculated at an energy of 40 keV. An experienced radiation oncologist used the PMI-70 dataset to delineate OARs for radiation treatments of head-and-neck and neuro-oncology cases, such as the brainstem, eyes, lenses, optic nerves and parotid glands.

### Automatic image segmentation methods

Two fundamentally different image segmentation methods are investigated; (i) an organ-driven MA method and (ii) a two-step 3D U-Net DL method. In 2017, both segmentation methods were evaluated with state-of-the-art segmentation methods in the AAPM thoracic auto-segmentation challenge^19^. Due to the limited patient cohort of 14 subjects, the leave-one-out cross-validation approach was applied to test the general performance of both image segmentation algorithms on multiple PMI datasets. The MA method is applied on all calculated PMI datasets (N = 7). The 3D U-Net method is, due to practical restrictions (GPU calculation time), only applied to PMI datasets of the reference energy (70 keV) and the energy, which provided the best results for the MA method.

#### Organ-driven multi-atlas based image segmentation

The in-house developed algorithm for MA based image segmentation^20^ consists of three major steps: the pre-processing, the deformation and the post-processing step. The atlas database is composed of atlases with its CT and OAR segmentation volume. In the pre-processing step, the CT volume is denoised using the edge-preserving multi-threaded curvature flow image filter (5 iterations; time step = 0.05) implemented in the Insight Segmentation and Registration Toolkit (ITK).

In the deformation step, Elastix was used to apply a multi-stage deformable image registration between the CT volume of the unsegmented patient and the CT volumes stored in the atlas database^21^. This deformable image registration algorithm first calculates an affine transformation followed by a B-spline transformation. The calculated deformation field is then used to deform the CT and segmentation volume from all atlases within the atlas database to the unsegmented CT volume. For every OAR, a consensus between the deformed segmentation volumes is found by applying the simultaneous truth and performance level estimation (STAPLE) filter on the atlases with the highest normalized cross-correlation (NCC) coefficient around the OAR^22^. In the final post-processing step, the segmentations resulting from the STAPLE filter were morphologically smoothed to obtain the final segmentation. The method was processed on a 400 core HTCondor CPU cluster.

#### Two-step 3D U-Net deep-learning method

The two-step 3D U-Net DL method is a 3D convolutional neural network architecture, which was applied twice in succession. First, the 3D bounding box location of each OAR was detected, and second, the OAR found in the previously detected bounding box was segmented.

As a result of the AAPM 2017 thoracic auto-contouring challenge^19^, the original implementation of the deep-learning (DL) algorithm, used in this study, was made available by Dr. Xue Feng from the University of Virginia. The algorithm was written in Tensorflow (Python 2.7) and made use of the NVIDIA’s CUDA® Deep Neural Network library (cuDNN) computational kernels.

To train the 3D DL convolutional neural network, a training dataset of atlases was created. Every atlas in the training dataset consisted of two 3D volumes: (i) the CT volume and (ii) the segmentation volume. Before learning the first step of the 3D U-net model, pre-processing of the CT and segmentation volume was performed. The segmentation volume contains information about the manual OAR segmentations wherein unique OAR flag IDs were assigned to every voxel in the volume. In this automatic pre-processing step, CT numbers between −500 HU and 1500 HU were normalized and both volumes (i and ii) were resized to unify voxel dimensions (0.98 × 0.98 × 2.00 mm³).

The first training step (900 epochs) was applied on down-sampled and cropped volumes. The normalized CT and segmentation volume were down-sampled to half of its original dimensions, where after the volume was cropped with 48 voxels to remove less important air voxels from both volumes. Unique OAR flag IDs were assigned in the segmentation volume, except for the eye lenses. No model was learned for the eye lens, because its volume was too small after down-sampling. Alternatively, the segmentation volume of the eye lens was morphologically subtracted from the segmentation volume of the eyes before down-sampling.

The network architecture contained three encoding and three decoding layers, used weighted cross entropy as loss function and the dropout was equal to 0.5. For each OAR, a bounding box with preset fixed sizes was determined. The bounding box size was equal to [80, 88, 88] voxels for the brainstem, [48, 88, 88] for the eyes and optic nerves, and [64, 120, 120] for the parotid glands. In the second step, one network (500 epochs) is trained per OAR to segment the foreground pixels. In the last automatic post-processing step, the segmented foreground pixels of the OAR were cleaned. The non-contiguous regions were removed and the binary holes were filled using morphological operations. Because no model was trained for the eye lens, an algorithm was written to detect the binary hole in the eye segmentation automatically, and to identify these pixels as eye lens. In total, 28 neural networks were trained and applied on two graphics cards: the Geforce Titan Xp and the Quadro P6000.

### Quantitative and qualitative segmentation accuracy assessment

For quantitative assessment of the image segmentation accuracy, three evaluation metrics were used: the Dice similarity coefficient (DSC), the 95^th^ percentile Hausdorff distance (HD), and the center of mass displacement ($\triangle {CoM}$). Each metric compares the automatic segmentation ${S}_{{AUTO}}$ and the manual delineation performed by the radiation oncologist ${S}_{{RO}}$, which serves as ground truth here. The DSC calculates the overlap between two 3D volumes and is equal to one for a perfect overlap and equal to zero without any overlap. The distance between two outer surfaces is described by the HD, where the optimal outcome is equal to 0 mm and where increasing distances indicate less or no overlap^23^. To ignore the influence of a very small subset of inaccuracies in the automatically generated OAR segmentation, the 95^th^ percentile of the HD is considered in this study. The Cartesian $\triangle {CoM}$ between two segmentations is a measure of the 3D position shift of the segmentation.

For qualitative assessment, two experienced radiation oncologists as well as an experienced radiation technologist individually scored the automatic image segmentation according to a discrete four-grade scale. The evaluation was done for all OARs on the PMI-70 dataset and additionally on the PMI-40 dataset for the optic nerves. A score of 1 was assigned to clinically unacceptable contours, for which it would take more time to modify than to restart a manual delineation. Score 2 or 3 were assigned to clinically acceptable contours with major and minor changes, respectively. A score of 4 was assigned to clinically acceptable contours with none or negligible changes. This qualitative assessment was performed twice on all 14 patients.

## Results

### Quantitative assessment

**Figure 2:**
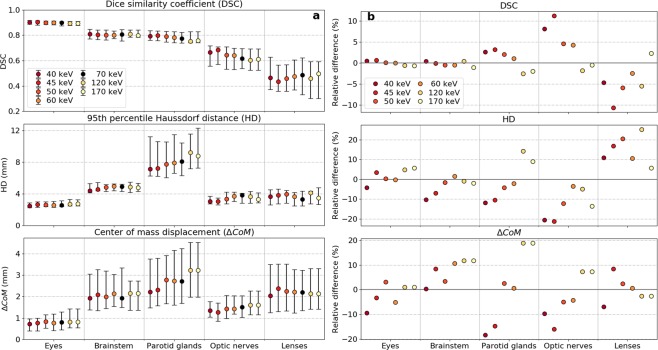
(**a**) Quantitative evaluation metrics calculated between the manual reference contour and the automatic segmentations generated by the multi-atlas based image segmentation using pseudo-monoenergetic image (PMI) datasets of 7 different energies ranging from 40 keV to 170 keV. The markers indicate the median value, the whiskers represent the 25^th^ and 75^th^ percentile and the black marker is the reference energy (70 keV). (**b**) Relative differences between PMI datasets of different energies and the PMI of the reference energy (70 keV).

The results of the three different evaluation metrics (DSC, HD, and $\triangle {CoM}$) for the MA method applied on various PMI datasets are presented in Fig. 2.

As the PMI-70 dataset was originally used by the radiation oncologist for manual contouring, it was defined as reference PMI in the further analysis. In general, the DSC between the manual and automatic contour was maximal when the MA method used PMI datasets of the lowest energy (i.e. PMI-40). The largest mean differences were found to be 2.6% for parotid glands and 8.1% for optic nerves. Smaller relative DSC differences were noticeable for brainstem (0.4%) and eyes (0.4%). In general, the HD between manual and automatic segmentation reduced on average when the MA method was applied on the PMI-40 dataset compared to the PMI-70 dataset: 10.4% for brainstem, 4.2% for eyes, 20.6% for optic nerves and 11.9% for parotid glands. A similar trend was observable for the $\triangle {CoM}$ metric. The DSC for eye lenses were difficult to interpret due to the large discrepancies over the whole energy range of PMI datasets.

**Figure 3:**
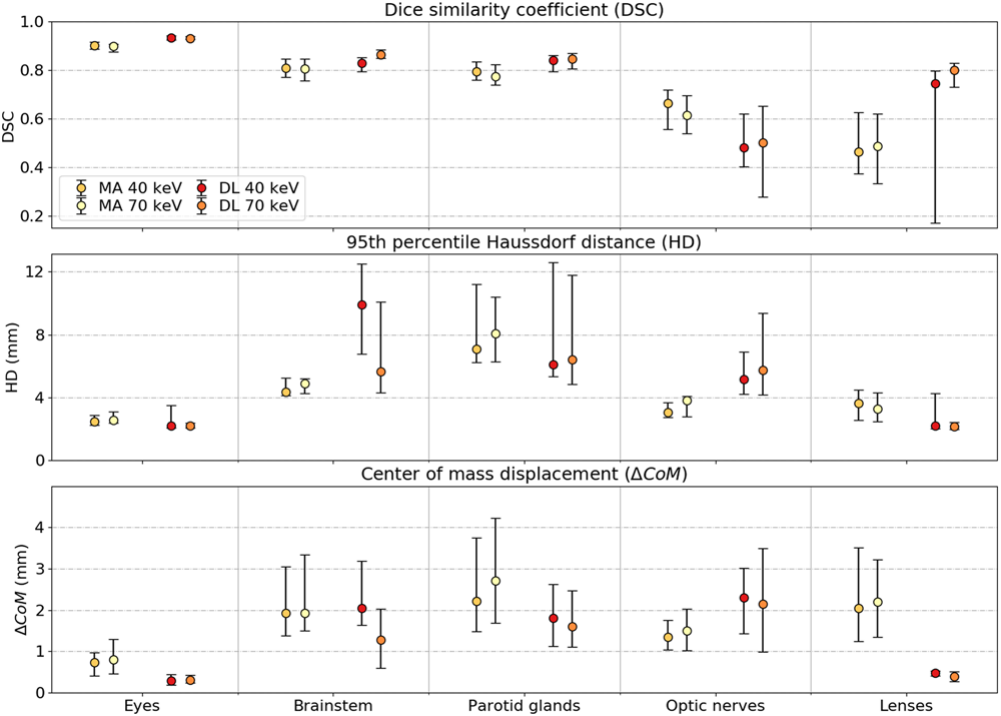
Quantitative evaluation metrics between the manual and automatic segmentations derived from pseudo-monoenergetic image (PMI) datasets of 40 keV and 70 keV for the multi-atlas (MA) and deep-learning (DL) based image segmentation. The markers indicate the median value and the whiskers represent the 25^th^ and 75^th^ percentile.

Considering the general improvements in DSC, HD, and $\triangle {CoM}$ for the MA algorithm using the PMI-40 dataset, the 3D U-Net DL model was trained on the PMI-40 and PMI-70 datasets in a leave-one-out cross-validation approach. Figure 3 depicts the three evaluation metrics for both segmentation methods using the PMI-40 and PMI-70 datasets.

The DL neural networks trained on the PMI-40 dataset slightly underperformed compared to the one trained on the PMI-70 dataset. The DSC reduced on average with 4.0% for brainstem and 4.1% for optic nerves. Smaller relative DSC differences between the application on PMI-40 and PMI-70 datasets were noticeable for eyes (−0.2%) and parotid glands (−0.7%).

The DL approach outperforms the MA method on all three quantitative metrics for both PMI-40 and PMI-70 datasets, except for optic nerves. Compared to the DL method, the DSC for optic nerves was on average 28% and 19% larger for the MA method applied on PMI-40 and PMI-70 datasets, respectively.

### Qualitative assessment

**Figure 4:**
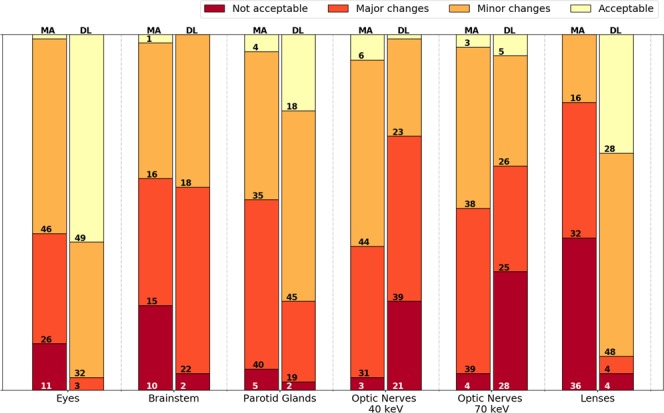
Stacked bar chart of the qualitative four-grade scoring (not clinically acceptable, clinically acceptable with major changes, clinically acceptable with minor changes, clinically acceptable) of the automatic multi-atlas (MA) and deep-learning (DL) based image segmentations. The numbers in the bars indicate the occurrence in each category by the medical experts. The sum of the occurrence is equal to 84 for all organs (14 patients, 3 scorers and left/right), except for the brainstem (N = 42).

Comparing both segmentation approaches in a qualitative four-grade scoring system using the PMI-70 dataset, DL-based segmentations were less assigned to the ‘not clinically acceptable’ category, except for optic nerves (Fig. 4). The application of the DL approach led to not clinically acceptable assignments (score 1) in less than 5% of all brainstem, eye lens and parotid gland segmentations. DL-based contours of the brainstem, eye, eye lenses and parotid glands were classified as acceptable with or without minor changes (scores 3 and 4) in 42.9%, 96.4%, 90.5% and 75.0%, respectively.

**Figure 5:**
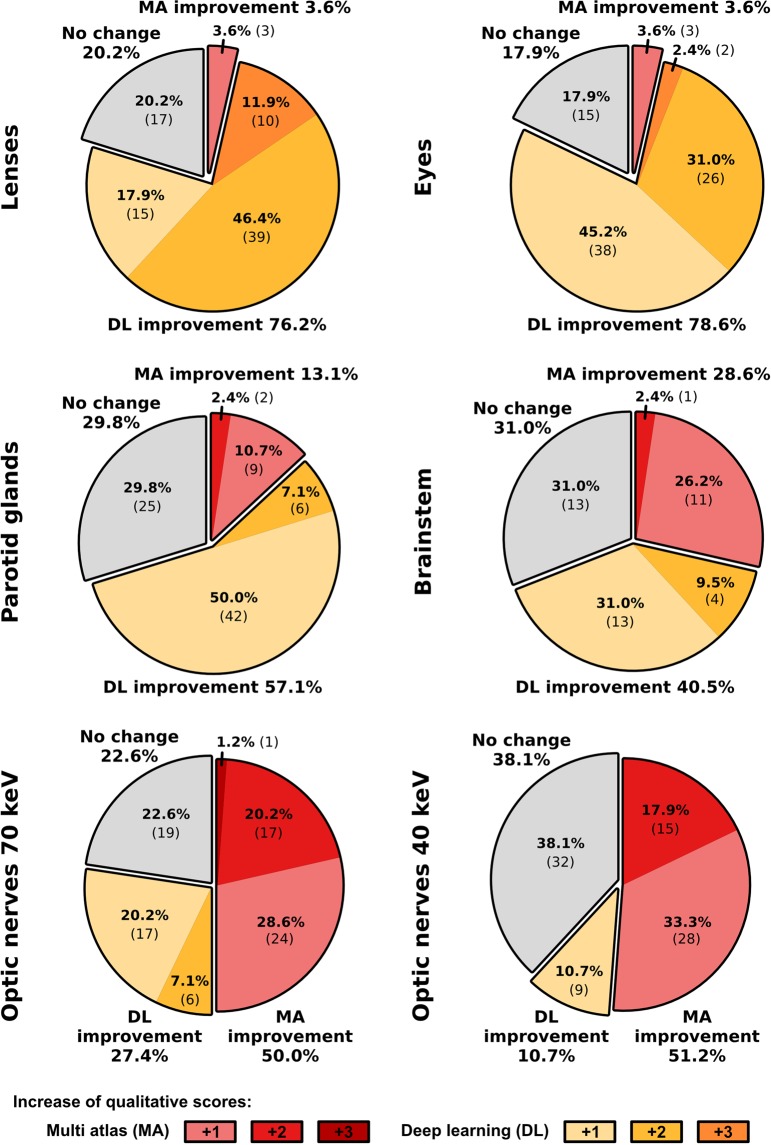
The relative and absolute occurrence of changes in the qualitative scoring between the multi-atlas (MA, red shaded) and deep-learning (DL, yellow shaded) methods including all observers. If the scoring of both approaches was the same, it was categorized as no change (grey shaded). For the respective method, improvements of one (light color) to three (dark color) qualitative scores were distinguished.

In accordance with the quantitative assessment, the optic nerve segmentations by the MA method scored better than the DL method (Fig. 5). Only 4.7% of the optic nerve segmentations using the MA method were assigned to the ‘not clinically acceptable’ category, where the DL approach resulted in 33.3%. The segmentations of the optic nerves using the PMI-40 datasets resulted in less assignments to the ‘major changes’ category and more assignments in the ‘minor changes’ category. The stacked bar charts of the three individual scorers are presented in the Supplementary Materials.

**Figure 6:**
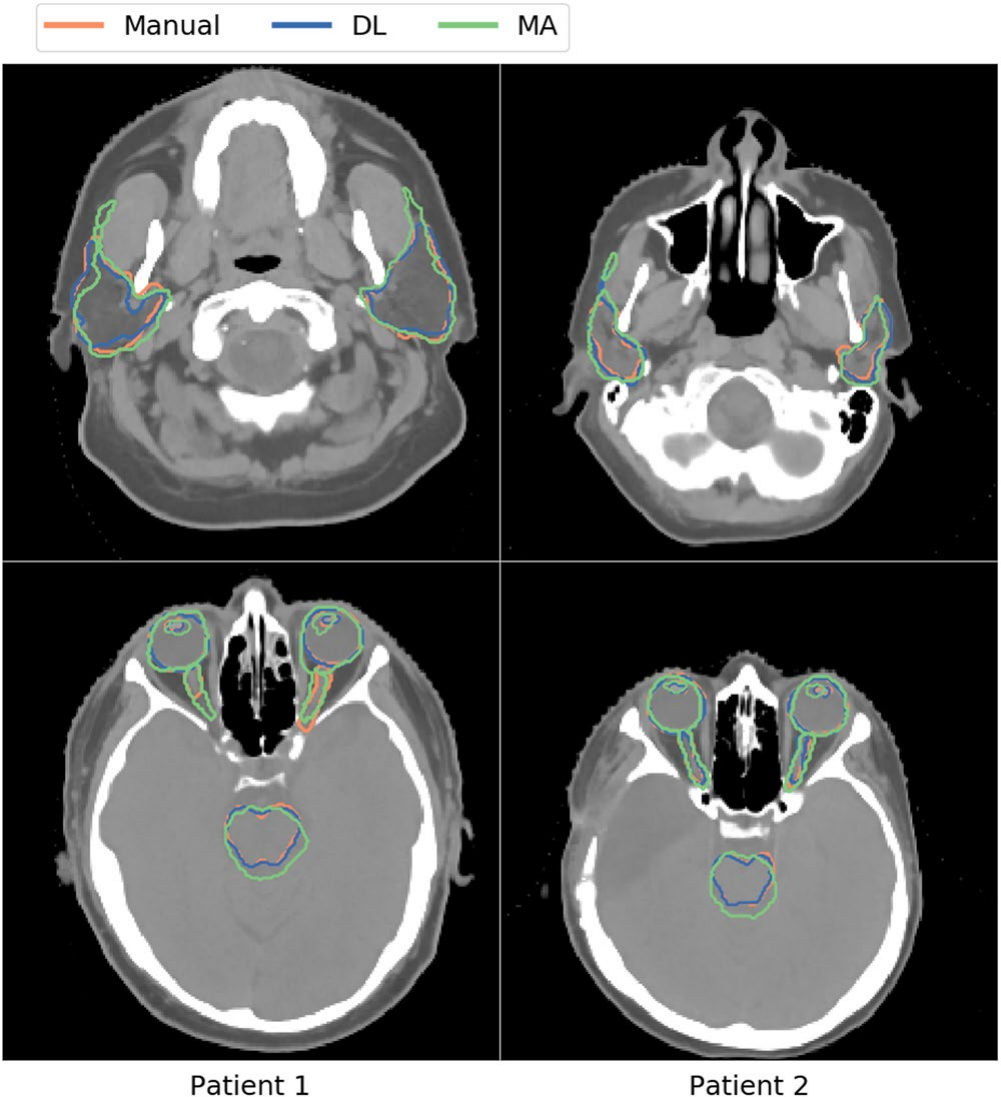
Comparison of the manual (orange), deep-learning (DL; blue), and multi-atlas (MA; green) based image segmentation methods for all organs-at-risk for two patients.

Figure 6 gives a visual representation of the manual and automatic OAR delineation for both segmentation methods. In the left column, patient 1 with the overall lowest DSC is shown. The right column shows patient 2 with the overall largest DSC.

### Calculation time

On average, the DL method demanded for training (53 ± 4) hours on a Geforce Titan Xp or Quadro P6000 (NVIDIA Corporation, Santa Clara, USA) for one neural network in our adopted leave-one-out cross validation. An average inference time of (20 ± 2) seconds was required to segment all OAR foreground pixels of one patient. The MA method which was performed on a 400 core HTCondor CPU cluster required around 10 minutes to segment all OAR foreground pixels of one patient.

## Discussion

Much effort has been clinically made to study the advantages of DECT imaging in radiotherapy, to demonstrate its clinical relevance and accuracy in dose calculation especially for proton therapy as well as to implement DECT in a clinical workflow^16,24–26^. However, to our knowledge, no study has been investigated automatic OAR segmentation methods on multiple PMIs, neither for MA methods nor DL approaches.

The MA and DL methods were quantitatively and qualitatively evaluated for various PMI datasets. Compared to the higher energies, for most OARs, the automatic segmentation using the MA method revealed better quantitative results using the PMI-40 dataset. This resulted in a DSC improvement of 8% for the optic nerves compared to the automatic segmentation generated using the PMI-70 dataset.

The PMI-70 dataset was also used for manual delineation. According to Wohlfahrt *et al*.^18^, this dataset reveals the best image-noise and contrast-noise ratio compared to the other six reconstructed PMIs. Since this might have introduced a small bias in the results, the quantitative metric errors of the MA method were reported relatively to PMI-70.

Thereafter, the PMI-40 and PMI-70 datasets were used for neural network training. In the quantitative results of the 3D U-Net, the contrary was observed. Here, the segmentation results of the PMI-70 dataset were slightly better than the segmentation results using the PMI-40 dataset, which may be explained by the lower noise levels in the PMI-70 compared with the PMI-40 dataset. To quantify differences in image noise between PMI-40 and PMI-70, the CT numbers in Hounsfield Units (HU) within a uniform brain region were evaluated in a circular region-of-interest, which were (50 ± 9) HU and (43 ± 5) HU, respectively. Except for the cerebrospinal fluid, the brainstem is surrounded by brain tissues having nearly the same image intensity and therefore it is more difficult to perform a segmentation while having higher noise levels in the non-contrast cranial body region.

The DSC, HD, and the $\triangle {CoM}$ metrics of the eye lenses were difficult to interpret due to the large discrepancies over the whole energy range. These discrepancies occurred because eye lenses are small and only consist of a few voxels. The inclusion or exclusion of a single voxel or only a few voxels will lead to a large change in the quantitative evaluation metrics.

Quantitatively and qualitatively, the 3D deep-learning approach performed better than the multi-atlas method, except for the optic nerves. The optic nerves are a relatively small volume delineated on every single axial CT slice. Since the anatomical extension of optic nerves is not only in transversal direction, relatively small volumes on multiple axial CT slices do not necessarily form a connected 3D object. This very likely caused the increased difficulties to find a contiguous segmentation volume of the 3D deep-learning approach compared to the MA method. An overview of the organ volumes is listed in the Supplementary Information.

The quantitative performance of both automatic segmentation methods was compared to published results of other methods. Zaffino *et al*.^27^ quantitatively assessed the performance of their automatic image segmentation software (PLASTIMATCH) applied on neurological cancer patients. They reported (values extracted using the DataThief3 software) a median DSC of 0.79 for brainstem, 0.43 for optic nerves and 0.78 for parotid glands using a mean of 18 ± 3.5 atlases to segment each patient. Considering the median DSC, our MA method applied on the PMI-40 dataset performed similar with 0.81 for brainstem, 0.67 for optic nerves, and 0.79 for parotid glands. Our DL approach applied on the PMI-70 dataset performed better for some OARs, such as 0.86 for brainstem and 0.85 for parotid glands. Here, a lower median DSC of 0.50 was calculated for optic nerves. Our DSC obtained for parotid glands, brainstem, and eyes using the DL method were comparable with the reported inter-observer variability of 0.85, 0.83, and 0.83, respectively^28,29^.

Considering the fact that both approaches, MA and DL, performed well for some OARs, while poorly for others (e.g. the optic nerves), more effort needs to be put into learning and evaluating the algorithms on larger datasets. However, the median DSCs reported in this study were comparable with the reported inter-observer variability assessed on conventionally used 120 kVp CT images^3,28,29^. Both algorithms were already compared to state-of-the-art automatic contouring techniques in the ‘thoracic auto-segmentation challenge’ organized at the 2017 annual meeting of the American Association of Physicists in Medicine, in further studies they should also be evaluated on other body sites^19^.

In terms of calculation time, the training time of the DL method is not the most important factor in OAR segmentation for radiotherapy, because the site-specific model training only needs to be performed once for a single DECT-derived PMI dataset of a specific energy and OAR combination. The time required to apply the automatic OAR segmentation on new patient datasets is more important in a clinical radiotherapy workflow, since the algorithm will be applied on each cancer patient being examined. The inference time of the DL method (±20 seconds) is much faster than the ±10 minutes of CPU cluster calculation time required by the MA method. Besides the improved OAR segmentation time of the DL method, the quantitative and the qualitative results were generally better for most of the OARs.

In this retrospective study, the automatic segmentations by state-of-the-art MA and DL approaches^19,20^ were evaluated between various PMI datasets (40 to 170 keV) on a limited atlas database of 14 primary brain-tumor patients that underwent DECT imaging to assess the influence of different image contrasts on delineation variability. Due to our adopted leave-one-out cross-validation approach, we do not expect a considerable change of the median value of the respective metric calculated for the MA method if more patients are included in this study. As a limitation of this retrospective study, the DL method was only trained on 13 patients (N-1), however, larger patient databases are desired for DL approaches.

Many automatic contouring algorithms in radiotherapy are still evaluated on conventional 120 kVp SECT images^19,30^. As already demonstrated in previous studies^10,18^, PMI datasets can improve image quality in terms of contrast and noise as well as reduce beam hardening artifacts depending on the energy selected for PMI generation compared to conventional 120 kVp SECT. For other applications such as a non-automatic and dedicated bone mineral segmentation, it has also been shown that the use of PMI datasets could reduce inter- and intra-observer variability^31^. However, to fulfill the requirements of the as-low-as-reasonably-achievable (ALARA) principle, only DECT scans were clinically acquired for the investigated patient cohort, since no added value for medical purposes was expected from a conventional 120 kVp SECT and an 80/140 kVp DECT scan in direct succession. Consequently, this retrospective study solely aimed at the evaluation of the performance of MA and DL methods on DECT-derived PMI datasets. After demonstrating the benefits of PMI datasets for automatic segmentation, a future prospective study could be designed to directly assess the differences between SECT and DECT considering the radiation protection law and an ethics approval.

## Conclusions

For the first time, a quantitative and qualitative assessment of multi-atlas and deep-learning based segmentation approaches was performed with DECT-based pseudo-monoenergetic images of different energies. For the multi-atlas method, PMIs calculated at lower energies generally resulted in better OAR segmentations. The neural network generally performed better than the multi-atlas method. However, the deep-learning approach encountered difficulties with the higher noise levels in PMIs of low energy. Further studies on larger image datasets and other body regions are needed to compare the performance of both OAR segmentation algorithms between DECT-derived datasets and possibly conventional 120 kVp SECT scans.

## Supplementary information

Supplementary Information
