# Supplementary material for: Dual-energy CT for automatic organs-at-risk segmentation in brain-tumor patients using a multi-atlas and deep-learning approach
Source: Sci Rep. 2019 Mar 11;9:4126. doi: 10.1038/s41598-019-40584-9 (PMC6411877; doi:10.1038/s41598-019-40584-9)
Supplement: Supplementary file 1 — Supplementary Information [file 41598_2019_40584_MOESM1_ESM.pdf]

SCIENTIFIC REPORTS

Dual-energy CT for automatic organs-at-risk segmentation in brain-tumor patients using a multi-atlas and deep-learning approach

Brent van der Heyden[1], Patrick Wohlfahrt[1], Daniëlle B.P. Eekers, Christian Richter, Karin Terhaag, Esther G.C. Troost[2], Frank Verhaegen[2*]

[1,2] These authors contributed equally.

Corresponding author: frank.verhaegen@maastro.nl

SUPPLEMENTARY INFORMATION

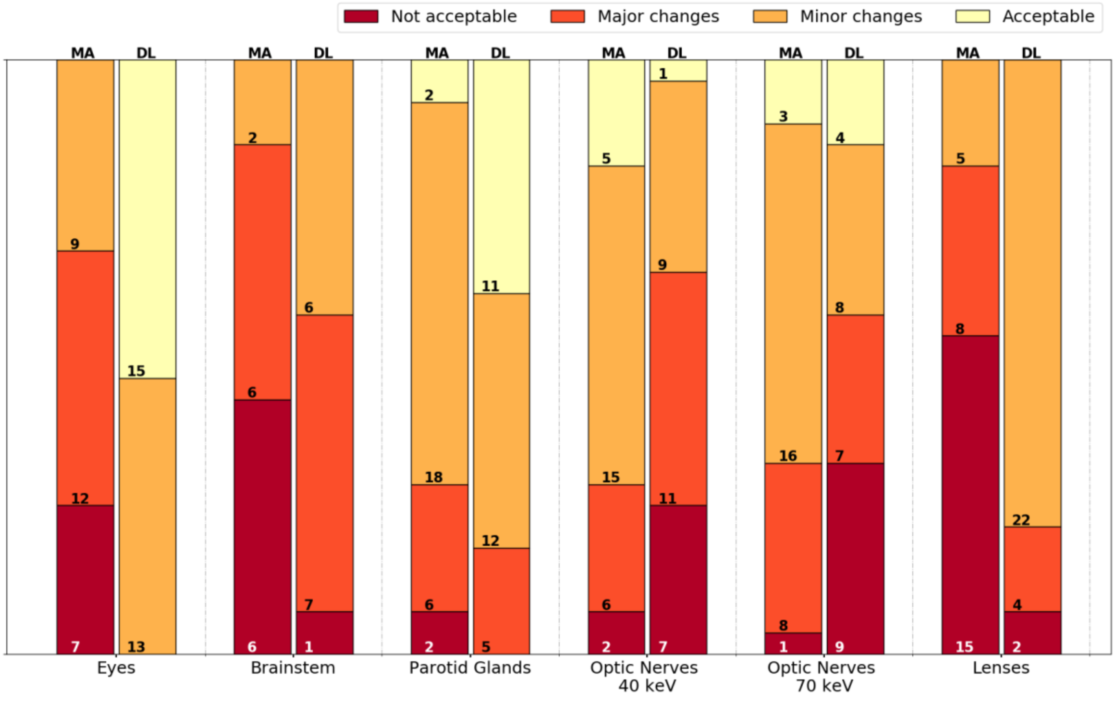

Figure S.1. Qualitative scoring by the first medical specialist.

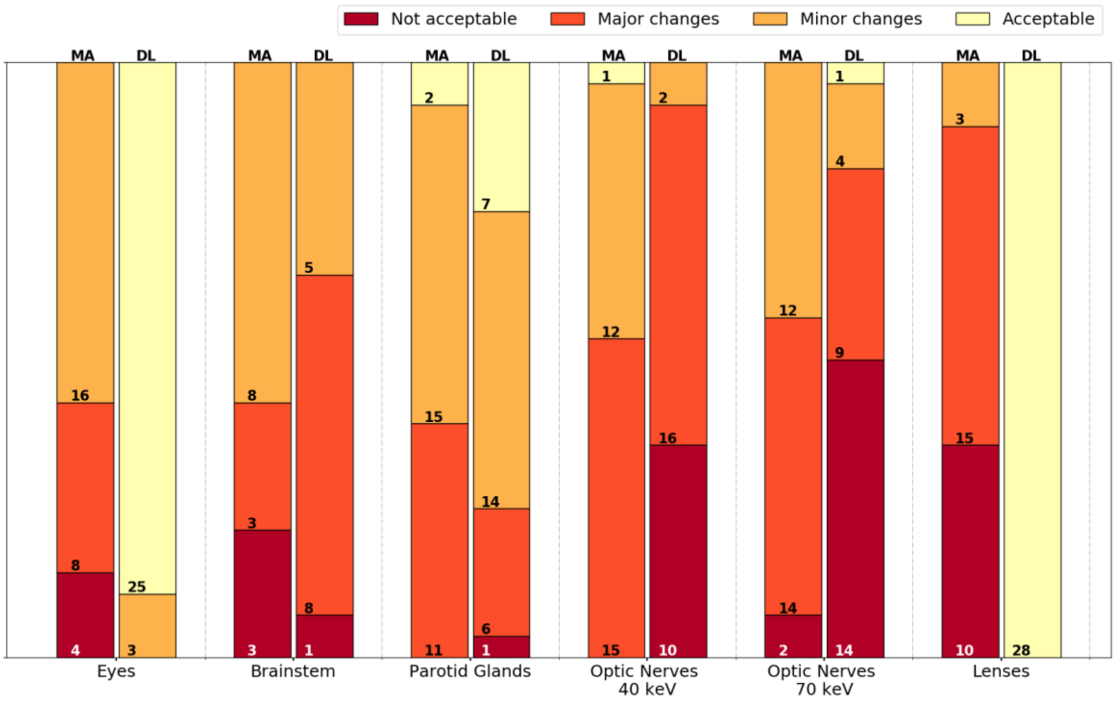

Figure S.2. Qualitative scoring by the second medical specialist.

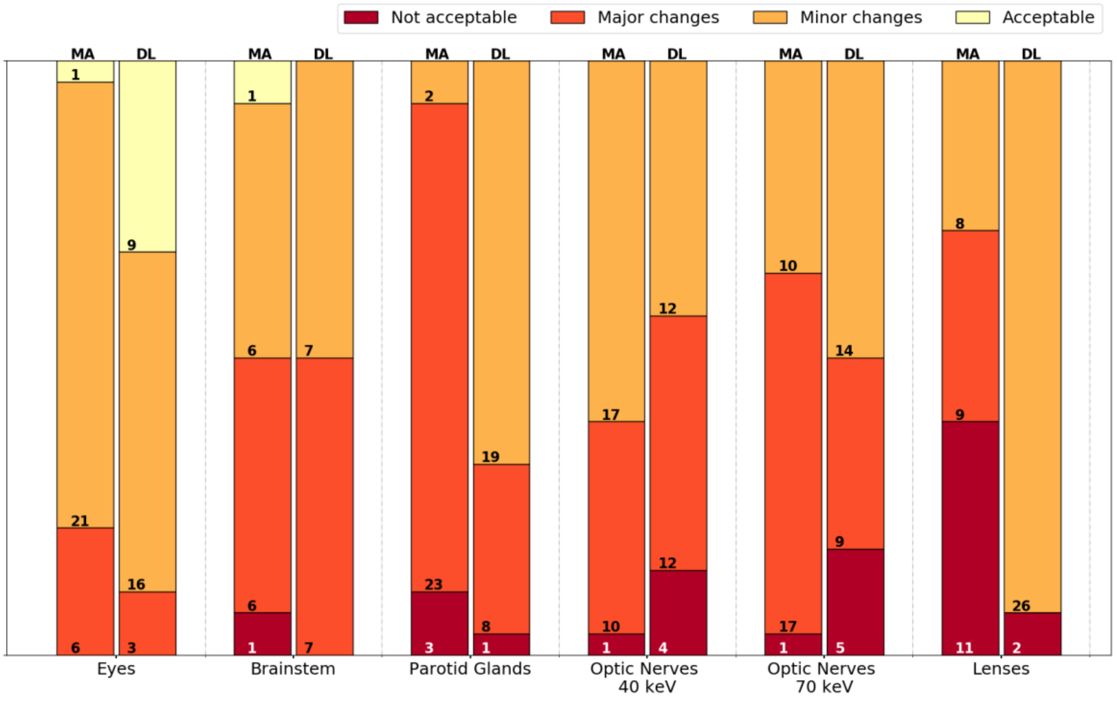

Figure S.3. Qualitative scoring by the third medical specialist.

SUPPLEMENTARY INFORMATION

The average organ-at-risk (OAR) volume was calculated for 14 patients. The volume is listed for five different segmentation methods: (i) manual segmentation, (ii) deep-learning based segmentation on the 40 keV image dataset, (iii) deep-learning based segmentation on the 70 keV image dataset, (iv) multi-atlas based segmentation on the 40 keV image dataset, and (v) multi-atlas based segmentation on the 70 keV dataset. The OAR volumes were determined considering the number of segmented voxels, the CT slice thickness, and the CT pixel spacing.

Appendix Table 1. Average organ-at-risk (OAR) volumes of the manual segmentation. (L = left, R = right, DL = deep-learning, MA = multi-atlas)

Average OAR volume [mm^3]

	**Manual**	**DL 40**	**DL 70**	**MA 40**	**MA 70**
Brainstem	23097	20015	22015	26766	26652
Eye L	7864	7518	7742	8037	8068
Eye R	8202	8013	8227	8393	8270
Lens L	183	166	132	217	240
Lens R	172	146	144	227	225
Optic Nerve L	524	295	295	665	681
Optic Nerve R	538	326	264	710	731
Parotid gland L	24040	21534	21301	28427	26974
Parotid gland R	24629	22349	21982	28820	27999

Standard deviation [mm^3]

	**Manual**	**DL 40**	**DL 70**	**MA 40**	**MA 70**
Brainstem	2647	4684	2757	4105	4756
Eye L	650	647	633	856	1106
Eye R	742	670	675	755	976
Lens L	30	85	57	77	113
Lens R	31	62	28	72	73
Optic Nerve L	159	155	233	122	134
Optic Nerve R	154	236	222	128	137
Parotid gland L	10392	9278	8237	12114	10938
Parotid gland R	10919	9165	8144	10340	9247

SUPPLEMENTARY INFORMATION

The optic nerves are a relatively small volume delineated on every single axial CT slice. Since the anatomical extension of optic nerves is not only in transversal direction, relatively small volumes on multiple axial CT slices do not necessarily form a connected 3D object. This very likely caused the increased difficulties to find a contiguous segmentation volume of the 3D deep-learning approach compared to the MA method. Following figures of the manually segmented optic nerves on three randomly chosen patients illustrate this statement.

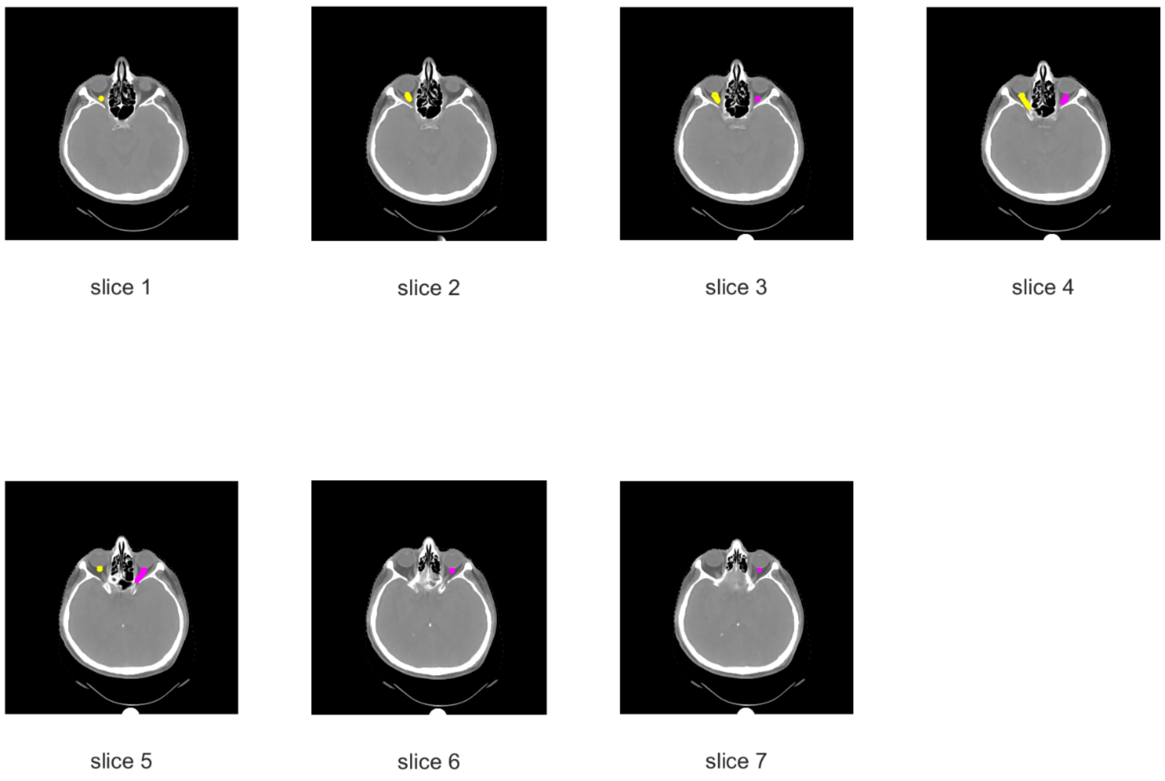

Figure S.4. Example patient 1

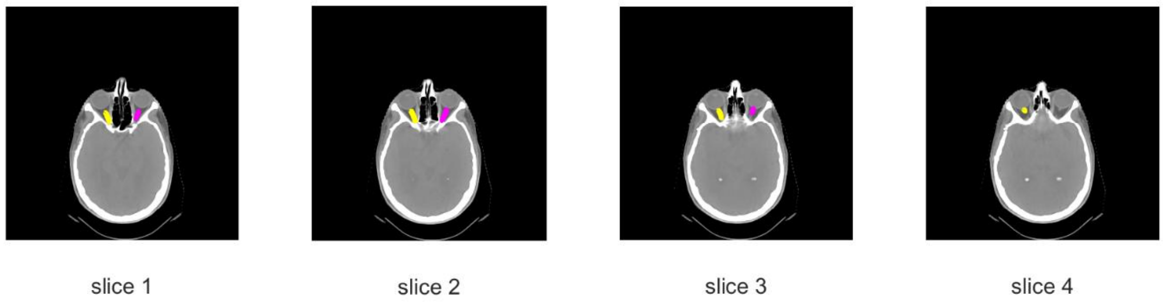

Figure S.5. Example patient 2

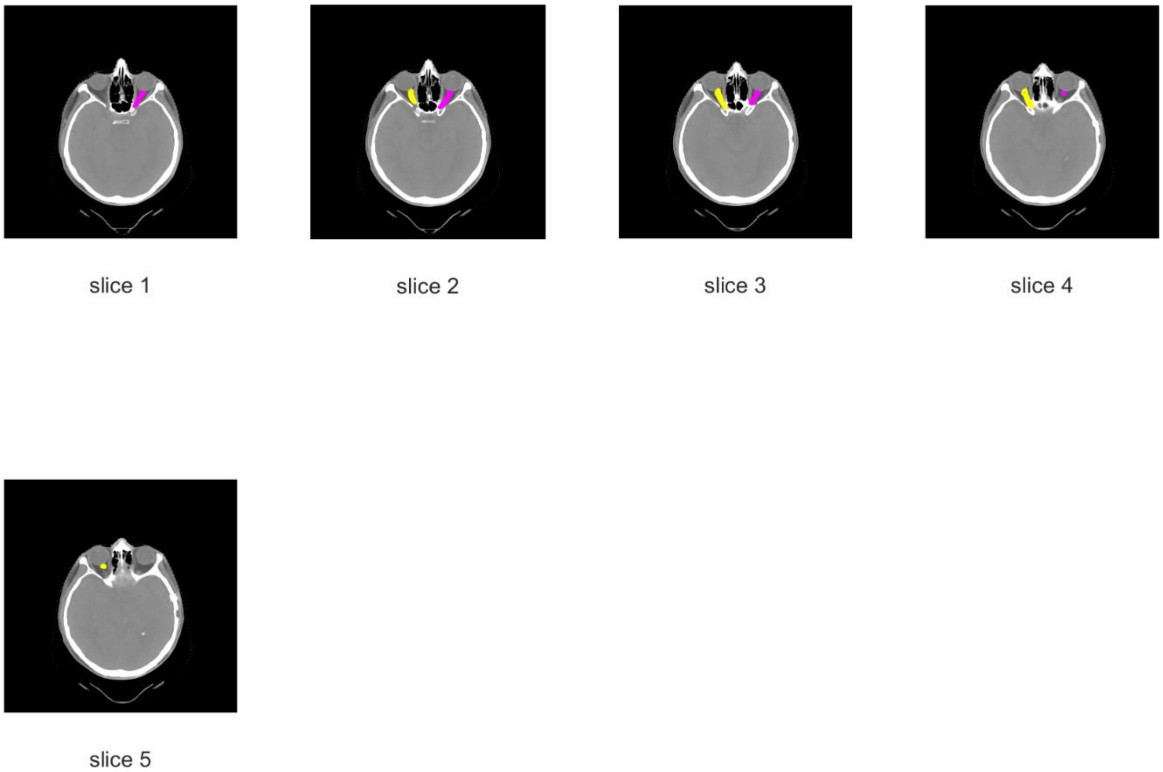

Figure S.6. Example patient 3
